# A Digital Toolkit (M-Healer) to Improve Care and Reduce Human Rights Abuses Against People With Mental Illness in West Africa: User-Centered Design, Development, and Usability Study

**DOI:** 10.2196/28526

**Published:** 2021-07-02

**Authors:** Dror Ben-Zeev, Suzanne Meller, Jaime Snyder, Dzifa A Attah, Liam Albright, Hoa Le, Seth M Asafo, Pamela Y Collins, Angela Ofori-Atta

**Affiliations:** 1 BRiTE Center Department of Psychiatry and Behavioral Sciences University of Washington Seattle, WA United States; 2 Information School University of Washington Seattle, WA United States; 3 Department of Psychiatry University of Ghana Legon Ghana; 4 Department of Global Health Department of Psychiatry and Behavioral Sciences University of Washington Seattle, WA United States

**Keywords:** mobile phone, low- and middle-income country, schizophrenia, bipolar disorder

## Abstract

**Background:**

The resources of West African mental health care systems are severely constrained, which contributes to significant unmet mental health needs. Consequently, people with psychiatric conditions often receive care from traditional and faith healers. Healers may use practices that constitute human rights violations, such as flogging, caging, forced fasting, and chaining.

**Objective:**

The aim of this study is to partner with healers in Ghana to develop a smartphone toolkit designed to support the dissemination of evidence-based psychosocial interventions and the strengthening of human rights awareness in the healer community.

**Methods:**

We conducted on-site observations and qualitative interviews with healers, a group co-design session, content development and prototype system build-out, and usability testing.

**Results:**

A total of 18 healers completed individual interviews. Participants reported on their understanding of the causes and treatments of mental illnesses. They identified situations in which they elect to use mechanical restraints and other coercive practices. Participants described an openness to using a smartphone-based app to help introduce them to alternative practices. A total of 12 healers participated in the co-design session. Of the 12 participants, 8 (67%) reported having a smartphone. Participants reported that they preferred spiritual guidance but that it was acceptable that M-Healer would provide mostly nonspiritual content. They provided suggestions for who should be depicted as the toolkit protagonist and ranked their preferred content delivery modality in the following order: live-action video, animated video, comic strip, and still images with text. Participants viewed *mood board* prototypes and rated their preferred visual design in the following order: religious theme, nature motif, community or medical, and Ghanaian culture. The content was organized into modules, including an introduction to the system, brief mental health interventions, verbal de-escalation strategies, guided relaxation techniques, and human rights training. Each module contained several scripted digital animation videos, with audio narration in English or Twi. The module menu was represented by touchscreen icons and a single word or phrase to maximize accessibility to users with limited literacy. In total, 12 participants completed the M-Healer usability testing. Participants commented that they liked the look and functionality of the app and understood the content. The participants reported that the information and displays were clear. They successfully navigated the app but identified several areas where usability could be enhanced. Posttesting usability measures indicated that participants found M-Healer to be feasible, acceptable, and usable.

**Conclusions:**

This study is the first to develop a digital mental health toolkit for healers in West Africa. Engaging healers in user-centered development produced an accessible and acceptable resource. Future field testing will determine whether M-Healer can improve healer practices and reduce human rights abuses.

## Introduction

### Background

At any given time, up to 6.8% of the world’s population has a serious mental illness (SMI), such as schizophrenia or bipolar disorder [1]. These psychiatric conditions are major causes of impairment and disability that produce particularly devastating long-term outcomes for individuals and communities with extreme resource constraints [2]. Individuals with SMI are at a heightened risk of homelessness, medical comorbidity, incarceration, victimization, and suicide [3]. In West Africa, the hardships of SMI are compounded by pervasive societal stigma, scarce treatment options, systematic exclusion, neglect, and abuse [4-7].

West African mental health care systems have severely constrained resources that contribute to significant unmet mental health needs [8-11]. Consequently, people with SMI who require mental health services often receive care from traditional and faith healers rather than from medically trained mental health professionals [12-14]. Ghana is a prime example of the disproportionate gap between the population’s mental health needs and access to mental health care providers trained in evidence-based practices: in 2017, the country had an estimated 27 million residents and approximately 6 psychiatrists and 7 psychologists for every 10 million residents, 3 psychiatric hospitals in total, and 4 community residential care facilities throughout the country [15]. Conversely, there are more than 44,000 traditional and faith healers in Ghana [9].

Ghana is one of the most religious countries on the planet [16]. The majority of the population in Ghana is Christian (ie, Pentecostal, Charismatic, and Evangelical), with the exception of the Northern region, which is predominantly Muslim [17]. Less than 6% of the population still follows the traditional religion, which expresses belief in both a supreme being and spiritual entities that reside in nature, but traditional shrines and remedies are still common and interwoven with other religious practices. Beyond economic and access barriers, many people seek out traditional and faith healers because they believe they will provide the type of care they or their family members are looking for [14]. Depending on the region’s dominant religion, an individual with mental illness may receive services from fetish priests, Christian pastors, or Muslim mallams [5]. Healers typically have no formal training in the etiology, diagnosis, or evidence-based treatment of mental illness. Traditional and faith-based care is not uniform and may vary dramatically across healers [12-14]. Healers often share the belief that psychopathology is spiritual in nature, and therefore, they may provide spiritual consultation, prescribe prayer, engage in sacrifices, or administer various ceremonial or herbal remedies [12-14,18,19]. In addition to these strategies that may have psychosocial benefits (eg, preaching helps address feelings of fear and offers patients a sense of hope and optimism [13]), healers may also use practices that constitute human rights abuses (eg, flogging, keeping patients in overcrowded enclosures or cages, forced fasting, shackling, exposure to the elements, and chaining patients to trees or concrete slabs for weeks or months) [7,18,19]. Chaining or similar forced mechanical restraining of people with SMI occurs in Africa, Asia, Europe, the Middle East, and the Americas [20]. These practices are psychologically damaging and physically dangerous [7,18-20].

Despite their oftentimes controversial practices, healers are the de facto providers and gatekeepers of care for people with SMI in West Africa [21,22]. Recent findings suggest that healers are open to engaging with mental health professionals, researchers, and technologists to learn about new and alternative approaches to managing the care of people with mental illness [23,24]. Our multinational, multidisciplinary research team has partnered with healers to develop and assess *M-Healer*, a digital toolkit designed to support the dissemination of evidence-based psychosocial interventions and the strengthening of human rights awareness and knowledge among healers in West Africa. The first stages of our development process focused on a *feasibility assessment*.

### Objectives

The primary objectives of this phase of the development process were to identify design requirements for a prototype M-Healer toolkit through off-site and on-site research activities, create the M-Healer prototype, and gather user feedback about the prototype from primary stakeholders in Ghana. On the basis of this foundational work, we concluded that (1) M-Healer would be most accessible if it was developed to be deployed via *smart* mobile devices with multimedia players that can deliver content in modalities other than written text for users with limited literacy; (2) content that undermines or negates the spiritual beliefs of the intended healer users or attempts to bypass them altogether (eg, directed at patients at the prayer camps while they are under the care of healers) would be counterproductive and may impede M-Healer adoption; and (3) in addition to the more typical illness-focused mental health intervention material that is typically used in mobile health (mHealth) treatment apps, there is also a crucial need to integrate content that focuses on the preservation of human rights, human dignity, and safety in practice [25]. Guided by these principles, we progressed to the next stages in the M-Healer user-centered design (UCD) and development process: individual interviews with healers, group co-design session, intervention content development and programming, and preliminary usability testing of a beta version of the M-Healer system with traditional and faith healers in West Africa.

## Methods

### Overview

The research reported here was approved by the institutional review boards of the University of Washington and the University of Ghana. Typical of digital health development work [26-29], the UCD, development, and testing procedures that produced M-Healer were segmented into distinct steps. Although the overall sequencing of the development process was predetermined, the outcomes of each step informed the specific content and rollout of activities in subsequent steps.

We conducted on-site interviews and observations at prayer camps in Ghana, a collaborative co-design session with healers to gather rapid feedback regarding possible design approaches, content development and prototype system build-out, and usability assessment with target end users. Interview protocols and elicitation materials drew on our previous work in Ghana [25], existing scientific literature on digital health and psychiatric services in West Africa, and user-centered technology design best practices from the field of information and communications technology for developing communities [30]. Study preparatory activities included conducting a comprehensive stakeholder analysis, researching digital infrastructure requirements, and developing participant interview materials. Our stakeholder analysis included identifying potential primary, secondary, and tertiary user groups; listing challenges of involving each of these stakeholders in on-site activities; and weighing anticipated costs and benefits of engaging with each stakeholder group. Digital infrastructure requirements focused on the likelihood of certain technologies being readily used, data and storage limitations, and other factors that would constrain future app development. We prepared visually enhanced data collection guides to help mitigate possible low literacy rates of the primary stakeholder population as well as potential language barriers between members of the research team and the faith healers. A reference library of descriptive photographs featuring African people and depicting a range of relevant situations, circumstances, relationships, and emotional states was created to use as needed when conducting interviews.

### Study Sites and Participants

Data collection activities were conducted with individuals who provided care to people with psychiatric illnesses in prayer camps in Ghana (ie, *healers*). Healers in the study varied in their position and rank within each camp and ranged from the most senior representative (ie, prayer camp leader or *prophet*) to more junior-level staff. All participants had regular direct interactions with patients in the camps. Individual interviews and usability testing activities were conducted with individuals at the camps where they worked. The co-design session involved a group discussion with participants from several different prayer camps and was therefore conducted in a neutral meeting center at a hotel in the region where all camps were located. Prayer camps varied in the number of patients with SMI they served at any given time, the number of staff members employed, and their specific service characteristics. All camps provided residential or inpatient care to people with psychiatric conditions. They range from well-appointed lodging with individual rooms to much more rustic communal settings. In one camp, patient stays were completely voluntary, and they could elect to leave at any time. At least two camps used observable forms of forced restraint and confinement. Shackles were observed in at least one camp, and locked enclosures were observed in another. Per participant reports, at least two camps used forced fasting as an intervention modality. All camps had centralized areas where communal prayer services were conducted daily and separate communal living areas where patients slept. Several camps had mattresses located in the main prayer area to accommodate *overflow* patients.

### Qualitative Interviews

Healer interviews were conducted by research team members who visited 3 prayer camps. Participants were compensated 50 Ghanaian cedi (approximately US $8.50) for their time. Camp representatives verbally indicated their willingness to participate in our research before the site visits and anticipated the team’s arrival. At the beginning of each site visit, the prayer camp leaders and staff were briefed about the nature of the project and the overall objectives of the research program. At all 3 prayer camps, healers offered a tour of the facilities to the research team either before or after the interviews. All healers present that day were invited to participate. Following informed consent, a research team member trained in qualitative interviewing strategies together with a team member fluent in Twi co-conducted the interview with a single participant in a private setting. Interview questions focused on healers’ current practices, needs, assessment of situations that lead to human rights abuses (eg, chaining, fasting, and forced seclusion), readiness to change practices, and interest in integrating technology to support their practices at the prayer camps. Interviews were audio recorded with permission and subsequently transcribed.

### Co-design Session

Healers were invited to participate in an upcoming co-design session. Interested individuals provided their contact information, and within a few days, a member of the research team followed up via telephone with an invitation and details. We convened a co-design session, including members of the research team and faith healers. The session was audio recorded and later transcribed. The objective of this session was to collectively ideate and assess the feasibility of the design directions for the M-Healer system. Before the session began, lunch was provided to the participants. Participants were compensated 100 Ghanaian cedi (approximately US $17) for their time. The session was cofacilitated by 1 Ghana-based (SMA) and 1 US-based researcher (DBZ), with materials developed by team members with expertise in participatory design (JS) and mHealth app content development (SM). Participants sat around a large table and were explicitly invited to share their experiences, opinions, and ideas. Following informed consent, participants were assigned a number (1-12) via an index card to facilitate rapid data collection. Facilitators referred to participants by this number, aiding in the proper attribution of speakers in transcripts. Participants were first introduced to mHealth and provided background information on the proposed mHealth technology. Participants were asked to evaluate and rate the aspects of the mHealth intervention model, intervention content, intervention modalities, and design preferences and rate them from 1 to 10 (0=strongly dislike and 10=like a lot). Participant numerical ratings directly informed the rapid synthesis of emergent design requirements and specifications.

### Content Development and System Design

M-Healer content was conceptually guided by the *stress-vulnerability model* [31] and *social rank theory* [32,33]*,* as they apply to the emergence and maintenance of psychopathology. The stress-vulnerability model posits that the course and outcomes of SMI are determined by the interplay of biological vulnerability, stress, and coping. To improve illness outcomes, illness management strategies based on this model aim to interrupt the cycle of stress and vulnerability that often lead to exacerbation of one’s condition [34,35]. Social rank theory suggests that pathological responses may emerge when vulnerable individuals find themselves in unwanted low status or rank positions with little ability to affect change or alter others’ perceptions of them. Feelings of shame and powerlessness are associated with the emergence, maintenance, and exacerbation of psychiatric conditions [36-39]. Furthermore, the use of restraints has been shown to worsen mood symptoms, hallucinations, and psychological distress and lengthen psychiatric inpatient stays [40-42]. M-Healer was created to guide healers on reducing their use of interventions that increase the stress placed on their patients (eg, mechanical restraints, fasting, isolation, and humiliation) and to train them on the use of simple psychosocial strategies to help patients better cope with their psychiatric symptoms in a manner that does not increase their feelings of shame and powerlessness.

M-Healer intervention content was initially developed by members of our team who are clinicians with expertise in providing care to people with SMI. Intervention module scripts were then circulated among all team members in the United States and Ghana to provide clinical input, lexical modification suggestions (ie, adopting terminology used in West Africa), and guidance on religious contextual anchoring to ensure that M-Healer content does not contradict or undermine prevailing beliefs in the region. Following appropriate modifications, we created a brief digital animation video depiction for each M-Healer intervention script in collaboration with a graphic artist. In parallel, we recorded spoken descriptions of each intervention in English and Twi (the most common languages in Ghana) by native Ghanaians. Audio recordings were then added to the digital animation videos so that users could select their preferred narration language for each intervention.

The M-Healer prototype development included building out a stable and self-contained Android platform, key path information architecture, integration of preliminary M-Healer branding, and examples of animated video and text content; the M-Healer user interface was developed in accordance with UCD principles and programmed to maximize accessibility and usability. We anticipate that M-Healer will be used in settings with low broadband support; therefore, the app was built to be self-contained with content delivered via optimized animated videos. Power-saving features, such as the dark mode, were integrated whenever possible.

### Usability Testing

User testing was conducted in Ghana in 2020, administered by the members of the research team based in Accra. The primary objective of prototype testing was to assess the perceived usefulness (value of the format and organization of content) and usability (ease of use of the interface and information architecture) of the M-Healer prototype. Faith healers who participated in the previous stages of the study were invited to provide feedback on the prototype. In addition, representatives of 2 additional camps participated in the testing. Participants were compensated 100 Ghanaian cedi (approximately US $17) for their time. Prayer camp leaders indicated their willingness to participate in our research before site visits and anticipated the team’s arrival. The research team members followed COVID-19 sanitation and social distancing practices as part of the research procedure. M-Healer was downloaded on team members’ Android smartphones and was used to introduce the tool to participants during testing. Following informed consent, a trained team member conducted a semistructured interview with a healer participant in their preferred language. Interviews were audio recorded in the participants’ preferred language and recorded in writing in English.

Participants were asked to interact with M-Healer for 2 minutes before initiating the interview. Participants were asked a series of open-ended questions about their experience navigating the tool, design preferences, and assessing content in 4 different domains: human rights content, relaxation content, verbal de-escalation content, and cognitive behavioral therapy skills training content. The research team focused on evaluating app content, interactivity, information design, and navigation.

Participants completed a 27-item measure comprising items drawn from a feasibility and acceptability questionnaire used in previous mHealth research [43] and items drawn from the user burden scale [44]. Participants also completed the 10-item system usability scale [45]. Participants were asked to rate their agreement with the measure statements using a 5-point bipolar rating scale (range 1=strongly disagree to 5=strongly agree).

## Results

### Qualitative Interviews

A total of 18 healers (16 males and 2 females) from 3 prayer camps completed individual interviews with our study team. The sample had an average age of 43 years (SD 11.2; range 26-66 years). All participants were Ghanaian. Self-identified titles ranged from *prophet* to *attendant* depending on roles, responsibilities, and position within the prayer camp organizational structure.

Participants reported their understanding of the causes and treatments of mental illnesses. Participants varied in their understanding of the etiology and treatment of mental illnesses. Most participants made clear distinctions between the conditions they believed were caused by spiritual problems that require spiritual or religious intervention and what they designated as mental illnesses with physical or biological origin that required medical intervention. They expressed confidence that they or the highest-ranking member of their prayer camp could accurately distinguish between these types of conditions. For example, one faith healer explained:

There are some that are caused by spirits. There are some that are physical and some problems that are spiritual. So those problems that are physical, when they come in and they pray for them, they refer them to the hospital.

Participants described situations in which patients with SMI become aggressive or violent. Participants reported a variety of strategies to manage these behaviors, including prayer, fasting, mechanical restraints, and seclusion. These treatments are often prescribed by a high-ranking member of the prayer camp, such as a prophet or other spiritual leader, and implemented by attendants. Most participants interviewed indicated that using mechanical restraints (ie, chaining) was a controversial practice. Recent exposure to government-sponsored campaigns to disincentivize such practices may have influenced their viewpoints; however, many participants stated that there were very few viable alternatives and that there was often no choice when a patient was aggressive or violent.

To better understand the factors that influenced whether patient behaviors were seen as resulting from spiritual or medical illness, we asked participants about the criteria they used when prescribing interventions such as forced fasting or restraints and how they assessed when to discontinue such interventions. Participants described how this was seldom a systematic process:

...A general reduction in the symptoms for which they were brought in, determines when they get out of chains. If somebody comes in shouting, suddenly he’ll quiet down and gain a certain level of consciousness that says-- why am I in chains? Because in the beginning they wouldn’t even know they were in chains and then when they get them out of the chains finally, they’re not acting out and they seem to follow instructions. There’s no specific criteria or specific things to look out for to get someone out of chains, but a general drop in the symptoms.

During the site visits, the team did not directly observe the chained patients. However, we did see a locked enclosure with patients at one of the sites and evidence of mechanical restraints in another, where unused shackles were connected to a wooden post in the main prayer or congregation area. When we inquired about the use of mechanical restraints, it became apparent that many healers were influenced by recent government campaigns, media reports, and advocacy efforts to reduce chaining practices. One participant described recent trends in reducing shackling:

...We stopped [using chains]...some people can be overzealous or overly aggressive that you can’t do much about...But now this has changed from the times where, from years ago the frequency would have been more compared to now.

In contrast, forced fasting was acknowledged to still be widely used. One healer explained that most patients entering the camp were expected to abstain from food according to a schedule determined by a healer:

Or sometimes if the person is a new person, a fresh person, when we see that the person is too aggressive we give them fasting. During the fasting the person will calm down. After 21 days, the person becomes sober.

Participants reported on their use of smartphones and their interest in a smartphone-based app in their daily work. When the research team showed an example of an mHealth app to participants, they were interested in the idea and at times endorsed the content displayed. One participant was now a pastor at a camp but previously was a patient who experienced auditory hallucinations. He described excitement hearing about an intervention to help people who hear voices and wanted to learn more about it.

### Co-design Session

A total of 12 participants (10 males and 2 females) from 3 prayer camps participated in the group co-design session. The sample had an average age of 45 years (SD 8.8; range 35-66 years). All participants were Ghanaian. All but one participant had participated in the previous qualitative interviews. In total, 67% (8/12) participants reported owning and using a smartphone.

Participants reported that they prefer spiritual guidance to medical guidance for working with patients. The research team indicated that they do not have the spiritual or religious expertise to assist in guiding those elements of their work and inquired whether it would be acceptable if M-Healer would provide mostly nonspiritual guidance, and all participants responded affirmatively. When asked who should deliver the M-Healer content or be depicted as a protagonist in the app, participants identified *pastors* (a term used for the higher ranking workers at the prayer camps) and doctors as the most trusted and credible source of information.

**Figure 1:**
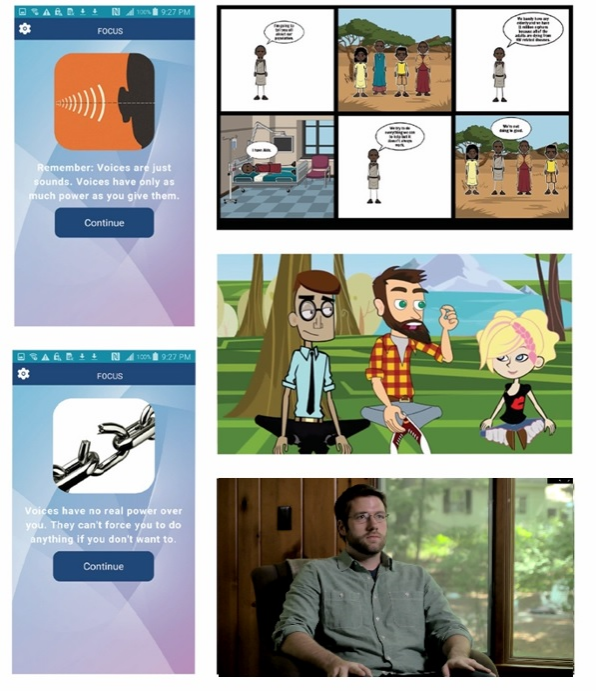
Presentation modes presented to participants during the co-design session. From upper left: 1, static text; 2, graphic novel style; 3, animated video; and 4, live-action video.

Participants were then shown a series of projected slides detailing different content delivery modalities: educational text with images, comic strips with text, animated digital videos with narration overlaid, and a live-action video discussing educational information (Figure 1). The live-action video was rated the highest (average score 9.5, SD 0.5), animated digital video was rated second (average score 6.7, SD 1.9), comic strip was rated third (average score 4.2, SD 2.6), and still images with text received the lowest rating (average score 2.8, SD 1.9). Although live-action videos were preferred overall, on further inquiry, participants reported no concern that animated content was too juvenile or made light of the topic.

**Figure 2:**
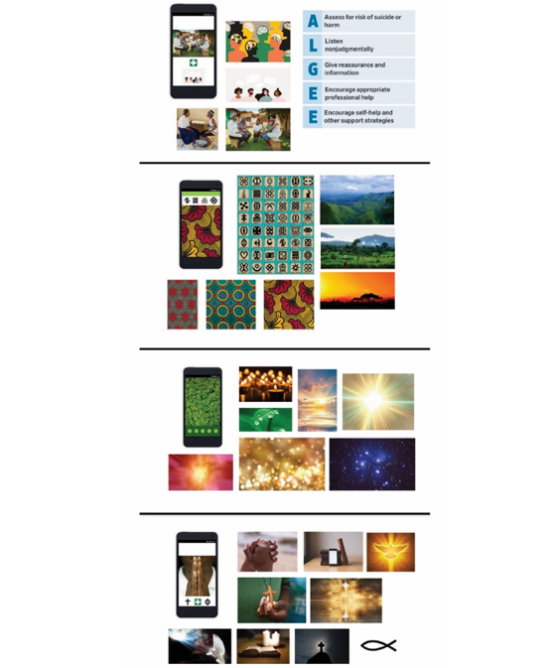
Four variations on visual motifs presented to participants during the co-design session. From top: community or medical, Ghanaian culture, nature motif, and explicitly religious.

Finally, participants viewed a series of 4 *mood board* visual prototypes to assess their preferences for the look and feel of app content and design (refer to Figure 2 for visual prototypes). The explicitly religious theme was rated highest (average score 9.3, SD 0.8), nature motif was rated second (average score 9.0, SD 0.9), community or medical was rated third (average score 6.9, SD 1.3), and Ghanaian culture was rated last (average score 5.0, SD 1.6).

### Content Development and System Design

M-Healer content is organized into modules, including an introduction to the system, brief mental health interventions, verbal de-escalation strategies, guided relaxation techniques, and human rights training. Each module contains several individually scripted digital animation videos that are approximately 1 minute long, with narration in English or Twi. The visual style of the app integrates spiritual motifs and metaphors without explicitly referencing any one particular religious iconography. M-Healer’s digital animations address healers as users and depict a healer or pastor figure as the main protagonist. Digital animations provide information on the physical harm; spiritual harm; and psychological effects of chaining, forced seclusion, and forced fasting. Combined with animations for training on psychosocial strategies, these tools are meant to increase healer awareness of their own decisions to use coercive practices and help reframe their own interpretations (ie, a loud or distressed patient is not necessarily going to act violently; if someone is hearing voices, they may be feeling afraid; and there is a high long-term cost to patients who experience chaining) and to offer viable alternative solutions.

**Figure 3:**
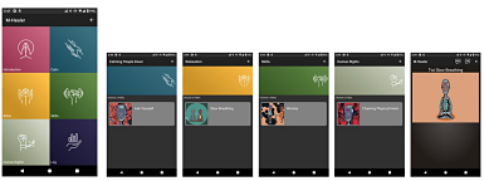
User interface of the prototype M-Healer app.

The M-Healer touchscreen user interface was designed and programmed to maximize accessibility and usability. The module menu is represented by touchscreen icons and a single word or phrase so that users with limited literacy would be able to navigate the system successfully based on visual depictions of the content of each module. The app is compatible with smartphones running Android operating system version 4.1 or higher (the majority of smartphones in West Africa) and can be made available for download from the Google Play store. M-Healer is navigated entirely by clicking on icons on the device touchscreen. The software enables flexible user-initiated demand access to all modules and digital animations via the home screen (Figure 3).

### Usability Testing

A total of 12 participants (11 male and 1 female) from 5 prayer camps participated in the M-Healer usability testing. The participants had an average age of 50 years (range 34-63 years). All participants were Ghanaian. Participants reported an average of 18 years of experience working with patients with mental illness in camps. Of this sample, 7 individuals participated in at least one of the previous study steps.

Overall, the open-ended feedback on the M-Healer app was positive. Participants commented that they liked the look and functionality of the app and understood the content. Participants reported that the information and display of the app were clear:

I am okay with the app. I feel the app contains information that could be useful. I am satisfied with animation and audio.

In total, 2 participants reported issues with the home screen icons representing different content areas; for example, one reported that the icons were confusing.

Participants were able to view, understand, and summarize clinical content from the animated videos shown during the usability testing: (1) calming down before working with aggressive patients (de-escalation), (2) deep breathing skills (relaxation), (3) skill for managing worried thoughts (cognitive intervention), and (4) harm of chaining practices on patient health (human rights). Animated video narrations were available in Twi or English, and all participants preferred the Twi language version:

I don’t know the breathing technique but think it might work. But I would need to practice to be able to do it effectively.

In addition, participants reflected on learning new concepts and skills that could be used in work with patients with SMI: “I did not know I could use this to help people get less aggressive.” Some participants indicated that the content was acceptable because some features incorporated spiritual values and practices: “I am happy about the use of prayer in the skills video.” Participants generally did not like the written text below the home screen module icons and preferred audio Twi content. The research team learned that although the local dialect is commonly spoken, it is not often read or written. Therefore, having an audio indicator associated with each module icon would enable greater usability and ease of content selection: “A voice to say what each tab is would be helpful.” Participant ratings of app feasibility and acceptability were promising, with the majority of participants rating the M-Healer app as intuitive, functional, and understandable (Table 1). Their system usability scale ratings (mean score 75, SD 12) indicate above-average usability [46].

**Table 1:** Participant ratings of M-Healer feasibility and acceptability.

Statement	Response	Participants, n (%)
“**I am satisfied with M-Healer.”**		
	Neutral	2 (17)
	Agree	5 (42)
	Strongly agree	5 (42)
“**I think that I would like to use M-Healer often.”**		
	Agree	7 (58)
	Strongly agree	5 (42)
“**It was easy to learn to use M-Healer.”**		
	Neutral	3 (25)
	Agree	7 (58)
	Strongly agree	2 (17)
“**I found M-Healer very awkward to use.”**		
	Strongly disagree	3 (25)
	Disagree	8 (67)
	Agree	1 (8)
“**I felt comfortable using M-Healer.”**		
	Agree	9 (75)
	Strongly agree	3 (25)
“**I found M-Healer to be very complicated.”**		
	Strongly disagree	2 (17)
	Disagree	7 (58)
	Neutral	3 (25)
“**I get frustrated when using M-Healer.”**		
	Strongly disagree	5 (42)
	Disagree	6 (50)
	Neutral	1 (8)
“**M-Healer is fun to use.”**		
	Neutral	1 (8)
	Agree	7 (58)
	Strongly agree	4 (33)
“**M-Healer works the way I want it to work.”**		
	Neutral	3 (25)
	Agree	6 (50)
	Strongly agree	3 (25)
“**It was easy to find the information I needed.”**		
	Disagree	2 (17)
	Neutral	1 (8)
	Agree	5 (42)
	Strongly agree	3 (25)
	No answer	1 (8)
“**I would imagine that most people would learn to use M-Healer very quickly.”**		
	Neutral	1 (8)
	Agree	9 (75)
	Strongly agree	2 (17)
“**M-Healer forces me to make changes to how I normally use smartphone apps.”**		
	Disagree	1 (8)
	Neutral	1 (8)
	Agree	8 (67)
	Strongly agree	1 (8)
	No answer	1 (8)
“**M-Healer demands too much mental effort.”**		
	Strongly disagree	5 (42)
	Disagree	6 (50)
	Agree	1 (8)
“**I need assistance from another person to use M-Healer.”**		
	Strongly disagree	3 (25)
	Disagree	3 (25)
	Neutral	2 (17)
	Agree	3 (25)
	Strongly agree	1 (8)
“**I found that the different parts of M-Healer work well together.”**		
	Neutral	2 (17)
	Agree	7 (58)
	Strongly agree	3 (25)
“**How things appeared on the screen was clear.”**		
	Agree	6 (50)
	Strongly agree	6 (50)
“**If I have access to M-Healer, I will use it.”**		
	Agree	4 (33)
	Strongly agree	8 (67)
“**I would recommend M-Healer to a friend.”**		
	Agree	6 (50)
	Strongly agree	6 (50)
“**I feel I need to have M-Healer.”**		
	Agree	6 (50)
	Strongly agree	6 (50)
“**Using M-Healer makes me feel like a bad person.”**		
	Strongly disagree	4 (33)
	Disagree	8 (67)
“**Overall, I am satisfied with how easy it is to use M-Healer.”**		
	Neutral	1 (8)
	Agree	9 (75)
	Strongly agree	2 (17)
“**The information provided for M-Healer was easy to understand.”**		
	Agree	8 (67)
	Strongly agree	4 (33)
“**Information, such as images and sounds, from M-Healer is hard to understand.”**		
	Strongly disagree	2 (25)
	Disagree	5 (42)
	Neutral	1 (8)
	Agree	3 (25)
	Strongly agree	1 (8)
“**M-Healer is not appropriate for my cultural background.”**		
	Strongly disagree	2 (17)
	Disagree	8 (67)
	Neutral	1 (8)
	Strongly agree	1 (8)
“**M-Healer requires me to remember too much information.”**		
	Strongly disagree	3 (25)
	Disagree	6 (50)
	Neutral	1 (8)
	Agree	2 (17)
“**M-Healer presents too much information at once.”**		
	Strongly disagree	3 (25)
	Disagree	8 (67)
	Agree	1 (8)
“**I am worried about what information gets shared by M-Healer (privacy).”**		
	Strongly disagree	4 (33)
	Disagree	7 (58)
	Neutral	1 (8)

## Discussion

### Principal Findings

Digital technology can play an important role in the management of psychiatric illnesses in low- and middle-income countries [47]. Our multinational team successfully developed and completed the preliminary testing of M-Healer, a novel mHealth intervention designed to be used by healers who provide care to people with psychiatric illnesses in West Africa. Healers and staff from several prayer camps in the region were engaged throughout our development process and provided valuable input and guidance on M-Healer design, functionality, and content. Our study findings suggest that healers found the M-Healer prototype to be feasible, acceptable, and usable. These promising findings set the stage for further development and deployment of M-Healer in the context of real-world services at prayer camps in West Africa.

This project makes several valuable contributions to the field. The technology we developed was specifically designed to be used by healers serving people with mental illnesses. To our knowledge, this constitutes the first attempt to develop a digital mental health intervention to be used by this population. M-Healer content depicts a healer character as the main protagonist in the app’s digital animations and incorporates generalized spiritual visual motifs that were rated as favorable by healers in our user-centered development process.

Globally, there is a growing awareness of the need to protect the human rights of people with mental illness [18]. The World Health Organization has developed a toolkit designed to educate leaders, health care professionals, administrators, and policy makers on the assessment and advancement of more humane mental health care [48]. The M-Healer technology developed in this study complements these efforts by addressing human rights issues in a manner that may be more suitable for community-based paraprofessionals and laypeople. M-Healer content is directed toward healers and prayer camp staff who make day-to-day decisions and is designed to discourage the use of chaining, forced fasting, and other coercive practices. The technology provides recommendations for alternative psychosocial intervention strategies, including verbal de-escalation, guided relaxation, and active listening. As a downloadable smartphone app, M-Healer could be disseminated efficiently in prayer camps and other informal practice settings, which often fall beyond the reach of formal educational campaigns or governmental oversight and regulation.

The iterative M-Healer development and usability testing process demonstrated that by leveraging simple design and digital animations rather than written content, mHealth technology can be made to be accessible to people with limited education, literacy, or familiarity with digital health tools. Our user testing demonstrated that M-Healer was navigable and understandable by the intended target audience. Our intention to use digital animations with overlaid narration (English or Twi) rather than videos depicting actors speaking local dialects was to facilitate greater flexibility and future opportunities for leveraging the same digital animations in other regions in Africa where healers operate by adding more narration options in different languages (eg, Yoruba and French).

The project adds to the growing literature showing that despite their differing conceptualizations of the causes of mental illness and appropriate treatments, when treated with respect and mutual appreciation, healers and paraprofessionals who subscribe to more spiritual models of psychopathology are open to collaboration with academic researchers and clinicians promoting Western notions of evidence-based care [22]. This study extends previous findings in West Africa involving the integration of spiritual practices with pharmacological approaches [21,23,24] and demonstrates that healers are also open to exploring the use of digital mental health tools that guide them on psychosocial approaches.

### Limitations and Future Work

This study had several limitations. First, although English is Ghana’s official language, some participants did not speak English or felt more comfortable communicating with our group in Twi. In such situations, members of the research team who are fluent in Twi assisted the English-speaking investigators conducting interviews and group discussions by translating the information bidirectionally, in real time. In the context of these dynamic interactions, some content might have been lost in translation or misinterpreted. Second, many of the healers involved in the study participated in more than one stage of data collection (eg, interviews, co-design sessions, and usability testing). Continuity in their participation was useful in our iterative-staged technology development. However, the disadvantage of such an approach is that most of the individuals who completed usability testing were not naïve to M-Healer concepts or content, and responses may have been affected by previous exposure. Similarly, the continuity in the relationship between our group and participants may have instilled in healers a sense of heightened commitment to the study and loyalty to the research team. We took active steps to overtly emphasize to participants that all forms of feedback are welcome and useful, and there were several instances when participants voiced their criticisms of proposed M-Healer ideas or concepts and suggested alternatives. Nevertheless, participant bias or social desirability effects may have influenced some of their responses. Future testing with novel participants will help determine whether fully naïve healers are similarly enthusiastic about the technology. Third, healers were notified ahead of time when the project team will be visiting their prayer camps. It is possible that prayer camp staff modified their practices (eg, unchained, clothed, or bathed patients) in preparation for these site visits. Finally, the study examined system use and collected subjective user evaluations but did not evaluate M-Healer’s effectiveness. Systematic clinical deployment of M-Healer and rigorous evaluation of its effects on healer behavior and patient outcomes will determine whether the technology is useful in enhancing healers’ knowledge of psychosocial interventions and alters their use of more controversial practices. Addressing healer beliefs and practices is one piece of a multifaceted puzzle that influences why, how, and from whom people with mental illness receive care in Ghana. Additional work with a wide range of stakeholders, from people with lived experience and their family members to policy makers, will help inform the development and implementation of multicompetent mental health reform strategies to improve care and reduce human rights violations in the region.

## Abbreviations

mHealth: mobile health
SMI: serious mental illness
UCD: user-centered design
